# Rheb Promotes Triglyceride Secretion and Ameliorates Diet-Induced Steatosis in the Liver

**DOI:** 10.3389/fcell.2022.808140

**Published:** 2022-03-16

**Authors:** Chongyangzi Du, Wanchun Yang, Zongyan Yu, Qiuyun Yuan, Dejiang Pang, Ping Tang, Wanxiang Jiang, Mina Chen, Bo Xiao

**Affiliations:** ^1^ Neuroscience and Metabolism Research, State Key Laboratory of Biotherapy, West China Hospital, Sichuan University, Chengdu, China; ^2^ Department of Neurosurgery, West China Hospital, Sichuan University, Chengdu, China; ^3^ Shenzhen Key Laboratory of Gene Regulation and Systems Biology, School of Life Sciences, Southern University of Science and Technology, Shenzhen, China; ^4^ Department of Biology, School of Life Sciences, Brain Research Center, Southern University of Science and Technology, Shenzhen, China

**Keywords:** Rheb, triglyceride, VLDL, hepatosteatosis, ATP

## Abstract

Hepatosteatosis, characterized by excessive accumulation of lipids in the liver, is a major health issue in modern society. Understanding how altered hepatic lipid metabolism/homeostasis causes hepatosteatosis helps to develop therapeutic interventions. Previous studies identify mitochondrial dysfunction as a contributor to hepatosteatosis. But, the molecular mechanisms of mitochondrial dysfunction leading to altered lipid metabolism remain incompletely understood. Our previous work shows that Rheb, a Ras-like small GTPase, not only activates mTORC1 but also promotes mitochondrial ATP production through pyruvate dehydrogenase (PDH). In this study, we further demonstrate that Rheb controls hepatic triglyceride secretion and reduces diet-induced lipid accumulation in a mouse liver. Genetic deletion of *Rheb* causes rapid and spontaneous steatosis in the liver, which is unexpected from the role of mTORC1 that enhances lipid synthesis, whereas Rheb transgene remarkably reduces diet-induced hepatosteatosis. Results suggest that the hepatosteatosis in *Rheb* KO is an outcome of impaired lipid secretion, which is linked to mitochondrial ATP production of hepatocytes. Our findings highlight an under-appreciated role of Rheb in the regulation of hepatic lipid secretion through mitochondrial energy production, with therapeutic implication.

## Introduction

The liver is a central hub for multiple metabolic events including lipid metabolism (Bechmann et al., 2012). Hepatic lipid metabolism/homeostasis is critical to maintain the whole-body lipid homeostasis in response to nutrient levels and the physiological status. Disruption of hepatic lipid homeostasis characterized by excessive triglyceride (TAG) accumulation in hepatocytes causes a clinical condition known as hepatosteatosis (Cohen et al., 2011). How hepatic lipid homeostasis is impaired in hepatosteatosis remains incompletely understood, which hinders the development of effective pharmacological therapies to manage hepatosteatosis.

The regulation of hepatic lipid metabolism is orchestrated through a complex network involving *de novo* lipogenesis (DNL) (Crescenzo et al., 2013), autophagy/lipophagy (Tanaka et al., 2016), and lipid secretion mediated by very-low-density lipoproteins (VLDLs) (Ye et al., 2009). Studies also suggest a delicate interplay between carbohydrate/glucose oxidation with lipid homeostasis (Morral et al., 2007). For example, progressive mitochondrial dysfunction impairs hepatic ATP production and contributes to obesity-associated hepatosteatosis (Rector et al., 2010; van Zutphen et al., 2016). How mitochondrial dysfunction is linked to dysregulated hepatic lipid metabolism is not well studied. Earlier studies show that mitochondrial pyruvate dehydrogenase (PDH) activity inhibits hepatic triglyceride and cholesterol biosynthesis (Ribes et al., 1979; Stacpoole et al., 1983) and ameliorates hepatosteatosis in animal models (Zhang et al., 2018). PDH, a metabolic gate keeper of the mitochondria, controls the production of acetyl-CoA, the flux of the TCA cycle (tricarboxylic acid cycle), and regulates cellular ATP production. Hepatic ATP production and availability could also play a role in the regulation of lipid metabolism, including lipid secretion (Musso et al., 2009). Therefore, PDH is best positioned to play a role in the crosstalk between hepatic ATP production and lipid metabolism.

Our recent work identifies Rheb (a Ras-like GTPase) as a general activator of mitochondrial PDH activity of multiple cell types, including neurons, neuroglia, and hepatocytes, with implications to cellular ATP production (Jia et al., 2021; Yang et al., 2021). Rheb is a small GTPase that functions as a direct and essential activator of mTORC1, a kinase complex that functions as a master regulator of cellular metabolism (Saxton and Sabatini 2017), growth, and differentiation (Delgoffe et al., 2011; Zou et al., 2011; Zou et al., 2014). mTORC1 is well known to regulate lipid metabolism/homeostasis through *de novo* lipid synthesis (Peterson et al., 2011). Systemic administration of the mTORC1 inhibitor rapamycin in patients causes lipidemia, suggesting that mTORC1 could also be involved in multiple aspects of lipid metabolism. However, the role of Rheb in activating PDH-mediated mitochondrial activity is independent of its role in activating mTORC1. In response to metabolic needs, Rheb is translocated into the mitochondrial matrix, where it binds PDH phosphatases (PDP1 and PDP2) and stabilizes the phosphatase activity toward PDH and thus keep PDH in the active state (Yang et al., 2021). The effect of Rheb on PDH activity is not related to its capacity of activating mTORC1, but the relative amount of Rheb protein is more relevant (Yang et al., 2021). All these findings suggest that Rheb could regulate hepatic lipid metabolism/homeostasis through mTORC1-dependenet and -independent mechanism.

To examine this hypothesis, we have assessed the lipid metabolism of the liver altered by hepatocyte-specific Rheb knockout (KO) and knock-in (KI) and revealed a critical role of Rheb in regulating hepatic lipid metabolism. The findings indicate that although Rheb may play a role in fatty acid synthesis through mTORC1, but its predominant role in lipid metabolism/homeostasis is to promote triglyceride secretion. This new role of Rheb in lipid homeostasis could involve both mTORC1-mediated and mTORC1-independent mitochondrial PDH activation.

## Materials and Methods

### Reagents

Oil Red O (a lysochrome diazo dye) for staining lipid droplets, the phosphatidylcholine kit, and FGF21 ELISA Kit were obtained from Abcam (ab146295, ab83377, and ab212160). The BODIPY dye for immunostaining of lipid droplets was purchased from Invitrogen (D3922). For VLDL detection, Triton WR-1339 was obtained from Selleck (S4578). The biochemical kits, including triglycerides, cholesterol, and ATP, were purchased from BioVision. All the other reagents were of the analytical reagent grade. For Western blotting, the following antibodies were obtained from Cell Signaling Technology, including anti-pS6K (Thr389), anti-pS6 (Ser240/244), anti-p4EBP1 (Thr37/46), anti-CCTα, anti-ACL, anti-PDI, anti-pAKT (Ser473), and anti-AKT. The Beta actin, GAPDH, and ApoB antibodies were from Millipore. The pPDH-E1α (Ser293) and PDH-E1α antibodies were from Abcam. The DGAT2 antibody was from Santa Cruz Biotechnology. The method of procurement of the Rheb antibody has been described previously (Zou et al., 2011).

### Animals

The mice used in this study, including *Rheb* floxed/floxed (*Rheb* f/f) and Rosa26-*Rheb(S16H)* K/+ mice (C57BL/6 × 129 genetic background), were described in our previous work (Zou et al., 2011). Briefly, Rheb f/f mice with the exon three of *Rheb* “floxed” were crossed with mice harboring the Cre recombinase under the control of the albumin-cre promoter. The myc-*Rheb (S16H)* cDNA was cloned into Rosa26 loci to generate Rosa26-*Rheb(S16H)* K/+ mice, which were also crossed with Alb-cre mice to overexpress constitutively active *Rheb* in the hepatocytes.

All the mice were kept in SPF conditions with standard housing conditions in a temperature-controlled environment with 12-h light/dark cycles and received normal diet (ND) or high-fat diet (HFD; OpenSource Diets D12492, 60% kcal from fat) and water *ad libitum*. *Rheb* KO mice (*Rheb* f/f; Alb-cre) with Ctrl littermates (*Rheb* f/f) were fed with normal diet. *Rheb* KI mice (S16H) (*Rheb* K/+; Alb-cre) with Ctrl littermates (*Rheb* K/+ or K/K) were fed with high-fat diet (HFD) for 8 weeks to induce hepatosteatosis. All mouse work was approved and performed in accordance with the Animal Care and Use Committee guidelines of West China Hospital, Sichuan University.

### Histological Analysis

To analyze lipid accumulation in the liver, formalin-fixed paraffin-embedded liver sections were stained with H&E, and OCT-embedded frozen liver sections were stained with Oil Red O, according to standard protocols and examined by microscopy (Lee et al., 2017). Three discontinuous liver sections were examined for each mouse. Images were collected by using OLYMPUS cellSens Dimension software (OLYMPUS BX63 microscope). Cryosections of the livers were stained with Bodipy 493/503 (0.1 μM) for 15 min for quantification of lipid droplets (Baiceanu et al., 2016).

### Transmission Electron Microscopy

To examine lipid droplets in the liver, 1-mm-thick liver slices from control and *Rheb* KO mice were fixed in 0.1 M 2.5% glutaraldehyde plus 2% paraformaldehyde solution at 4°C overnight and then post-fixed with 2% osmium tetroxide for 2 h in the dark. After dehydration in ethanol, the tissues were embedded in Epon for ultra-sectioning. Sample sections were stained with 2% uranyl acetate and 3% lead citrate and viewed on a FEI CM-12 transmission electron microscope.

### Western Blotting

Liver samples were lysed in the buffer containing 50 mM NaF, 10 mM Na_2_P_2_O_7_, 2% SDS with protease and phosphatase inhibitors. For assessing serum proteins, serum (10 μL) was added to 50 μL of 2×Laemmli sample buffer and 40 μL of RIPA buffer (with 0.5% sodium deoxycholate and 1 M urea). Then, equivalent amounts of proteins were separated by SDS-PAGE and transferred to a PVDF membrane. After blocking with 5% skim milk, the membrane was incubated with respective primary and secondary antibodies (see the aforementioned Reagents section), according to standard procedures. The secondary antibodies, including Goat anti-Rabbit IgG-HRP (Cat#31460) and Goat anti-Mouse IgG-HRP (Cat#31430), were obtained from Pierce. The blots were visualized by using a SuperSignal West Pico Chemiluminescent Substrate, developed by Clinx and quantified by ImageJ.

### RT-qPCR

For the qPCR assay, total mRNA was extracted from liver tissues using the RT-qPCR Kit (Foregene, Chengdu, China). Total mRNA obtained was reverse-transcribed into cDNA using a PrimeScript RT reagent Kit With gDNA Eraser. SYBR® Primix Ex Taq^TM^ II was applied to quantify PCR amplification. The mRNA levels of genes were normalized to *Actin*.

### Metabolite Measurements

Blood samples were collected, and blood serum was isolated by centrifugation and stored at-80°C for analysis. Serum triglyceride (Abcam) and cholesterol (BioVision) were measured by using commercial kits, and ALT and AST were measured by using an automatic biochemistry analyzer (BS-180, Mindray, Shenzhen, China). Serum FGF21 concentration was measured with a Mouse FGF21 ELISA Kit (Abcam).

Extracts from mouse livers were used for the assessment of triglyceride (Abcam), cholesterol (BioVision), and phosphatidylcholine (Abcam) contents using commercial kits, according to manufacturers’ instruction. For hepatocytes measurement, primary mouse hepatocytes were isolated from control and *Rheb* KO mice by using a collagenase perfusion method according to previous studies (Seglen 1976; Sakai et al., 2012).

VLDL secretion was determined by blocking VLDL catabolism using Triton WR-1339 (Borensztajn et al., 1976; Ye et al., 2009). Mice were injected with Triton WR-1339 (400 mg/kg in PBS) through tail veins. Serum was collected 4 h after Triton WR-1339 injection, and VLDL in mouse serum was determined using the ELISA Kit (H249, Nanjing Jiancheng Bioengineering Institute). The data were analyzed by using ELISAcalc software, and a logistic curve was used for fitting the model (four parameters).

### Lipidomics

Lipids were extracted from 20 mg of a mouse liver as previously described (Maillo et al., 2017). Briefly, samples were reconstituted in 300 μL of IPA/methanol (50:50) and were analyzed using the positive/negative ion–switching method and a Thermo Scientific Q Exactive plus mass spectrometry combined with a Dionex Ultimate 3,000 Rapid Separation LC (RSLC) system. A total of 2 μL of the sample was injected onto the C30 column held at 40°C. The binary solvent system (flow rate 0.35 ml/min) consisted of a mobile phase A containing acetonitrile-water (60:40) with 10 mM ammonium formate and a mobile phase B consisting of IPA/acetonitrile (90:10) with 10 mM ammonium formate. The gradient started from 40% B, reached 100% B in 25 min, returned back to the starting condition, and remained there for another 5 min. The data were collected on a data-dependent LC-MS/MS mode. The mass range was 150–1,800 Da. The spray voltage was 3.5kV. MS-DIAL 3.9 was used for the alignment and quantification of peaks. Peaks with total score≥80 and SN ≥ 5 were included for the following analysis. Compounds were excluded from the following analysis if their abundance data in more than 50% QC samples or all samples were missing values, or if the coefficient of variance of abundance data were over 0.2. The KNN algorithm was used to impute missing values in the remaining dataset. The abundance data of compounds were log-transformed and normalized between samples using median normalization. The Wilcoxon rank sum test was used to evaluate the significance of differences in compound abundance between samples. *p*-value less than 0.05 was considered as statistically significant.

### Gene Knockdown

To knock down *Rheb* in HEK293T cells, the following oligonucleotides encoding guide RNAs were cloned into the LV5-gRNACas9 vector: gRNA1: TAG​CTA​GGA​AAT​ACG​GTG​AC; gRNA2: CCG​GGC​AAG​TAA​GTG​ACC​TC. Briefly, 2×10^5^ cells were seeded into the wells of a six-well plate for gRNA transfections. Next, cells were selected with puromycin (2.5 μg/ml for 72 h) to eliminate untransfected cells. The cells were collected for subsequent Western blots.

### Quantification and Statistical Analysis

Data represent the mean value, and the error bars represent the standard error of the mean (S.E.M.). Statistical analysis was determined by using GraphPad Prism 6.0. The two-tailed Student’s *t* test and one-way ANOVA (followed by the Tukey or Dunnett post hoc test) were performed for statistical analysis. A *p*-value of <0.05 is considered as statistically significant. *p < 0.05, **p < 0.01, and ***p < 0.001.

## Results

### Hepatic Deletion of *Rheb* Causes Spontaneous Hepatosteatosis

**FIGURE 1:**
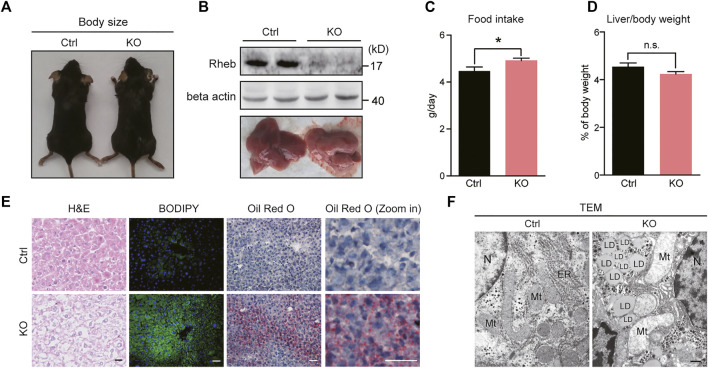
Hepatic deletion of *Rheb* causes spontaneous hepatosteatosis. **(A)** Body size of control and *Rheb* KO mice (6-week old). **(B)** Western blots of Rheb protein in the liver of control (Rheb f/f) and *Rheb* KO mice (Rheb f/f; Alb-cre); photographs of livers from control and *Rheb* KO mice (6-week old, lower panel). **(C)** Slightly increased food consumption of *Rheb* KO mice (control: n = 3, *Rheb* KO: *n* = 6). **(D)** The ratio of liver weight to body weight of control (n = 9) and *Rheb* KO mice (*n* = 10) (6-week-old). **(E)** H and E (left panel, scale bar, 20 μm), bodipy (middle panel, scale bar, 50 μm), and Oil Red O (right panel, scale bar, 50 μm) staining showing hepatosteatosis in the liver of *Rheb* KO mice (6-week-old). **(F)** TEM showing lipid droplets in the liver of *Rheb* KO mice (6-week-old). Scale bar, 1 μm. Data represent mean ± SEM. *p < 0.05. n.s. no statistical significance.

To determine the role of *Rheb* in lipid homeostasis, we genetically ablated *Rheb* in hepatocytes using albumin-Cre (hereafter *Rheb* KO) and asked how lipid homeostasis would be affected. As anticipated, albumin-cre–mediated deletion selectively reduced *Rheb* expression in the liver but not in the central or other peripheral organs, including the brain, small intestine, and pancreas (Figures 1A, B; Supplementary Figure S1A). *Rheb* KO slightly decreased the blood glucose level and increased food intake but did not alter body weight, liver/body weight compared to controls (Figures 1C,D, Supplementary Figure S1B, C).

To our surprise, the loss of *Rheb* resulted in a yellowish appearance of the liver (Figure 1B, lower panel). H&E staining (Figure 1E, left panel) revealed that the overall structural organization of the liver was normal, with signs of lipid accumulation in the hepatocytes. Lipid accumulation in the form of lipid droplets was revealed by BODIPY staining (Figure 1E, middle panel), indicative of hepatosteatosis. The progression of the hepatosteatosis was further monitored by performing Oil Red O staining, which showed that hepatosteatosis occurred acutely and spontaneously in *Rheb* KO mice fed with normal diet, and persisted throughout adulthood (Figure 1E, right panel, Supplementary Figure S1D). Finally, transmission electron microscopy (TEM) showed that lipid droplets are scattered in the hepatocytes of the *Rheb* KO liver (Figure 1F).

### Hepatic *Rheb* KO Impairs Triglyceride Secretion in the Liver

**FIGURE 2:**
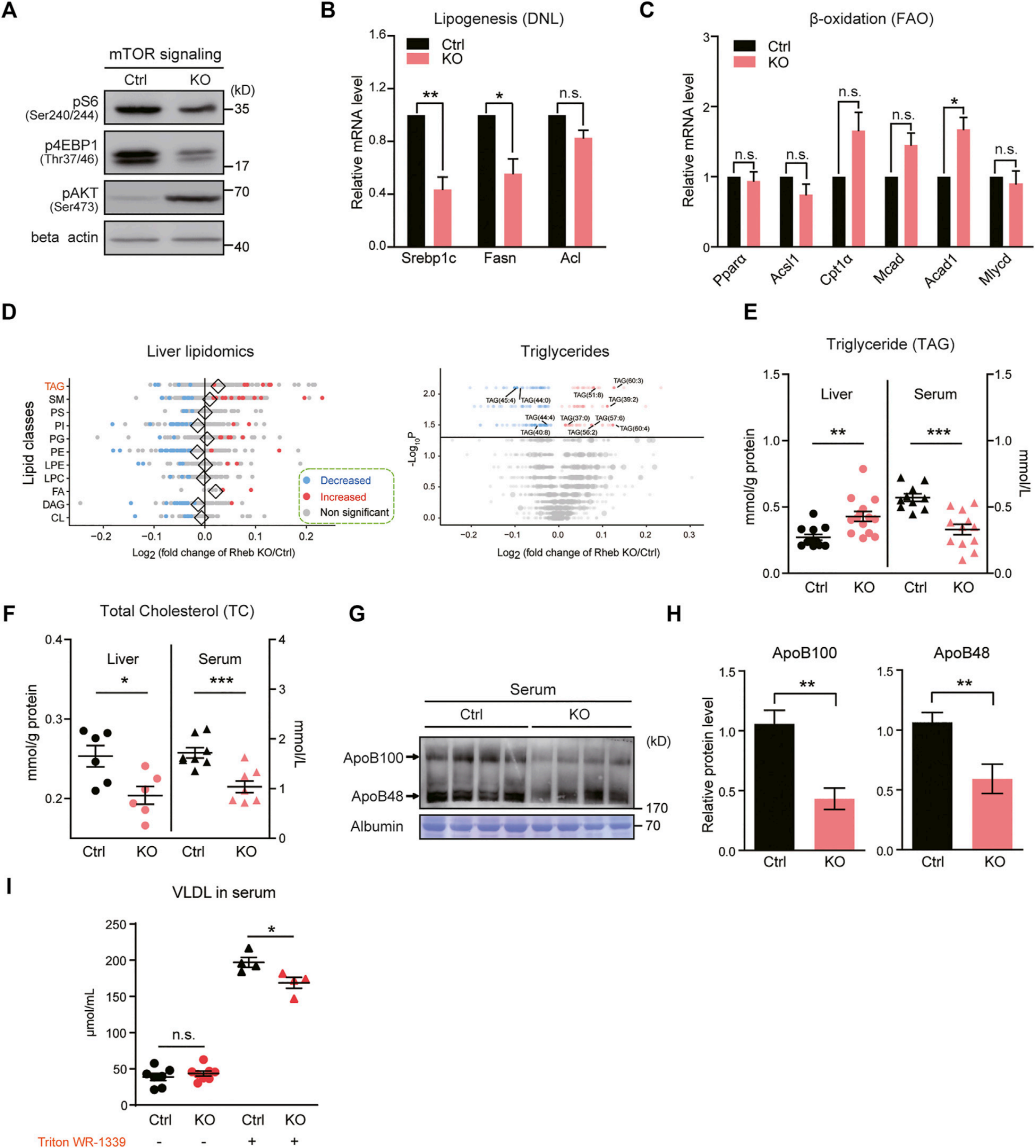
Hepatic *Rheb* KO impairs triglyceride secretion in the liver. **(A)** Western blots of mTORC1 activity in the liver of *Rheb* KO mice. **(B)** Quantitative RT–qPCR detection of lipogenic genes levels in the liver of *Rheb* KO mice (*n* = 4). **(C)** mRNA levels of fatty acid oxidation genes unchanged in the liver of *Rheb* KO mice (*n* = 4). **(D)** Forest plot showing the log fold changes of lipids in the liver of *Rheb* KO mice over controls. Red and blue points represent significantly increased and decreased lipids of each lipid class in the liver of *Rheb* KO mice. Diamonds indicate the average log fold change of lipids in each lipid class. Shown here are selected lipid classes. Volcano plot showing log fold changes and significant difference of identified lipids in the liver of *Rheb* KO mice. Total number of differential lipids is shown in the guide. Triglycerides are represented by larger points and significantly different triglycerides are annotated. **(E)** Scatter diagram showing an increased liver triglyceride level and decreased serum triglyceride level of *Rheb* KO mice (liver: *n* = 12, serum: *n* = 10). **(F)** Scatter diagram showing decreased liver and serum total cholesterol levels of *Rheb* KO mice. **(G,H)** Western blots **(G)** and quantifications **(H)** showing the decreased serum ApoB100 and ApoB48 levels of *Rheb* KO mice (*n* = 4, normalized against serum Albumin). **(I)** Diagrams showing the decreased VLDL levels in the serum of *Rheb* KO mice (*n* = 4). Data represent mean ± SEM. *p < 0.05, **p < 0.01, ***p < 0.001. n.s. no statistical significance.

The massive accumulation of lipid droplets in *Rheb* KO hepatocytes is unexpected because Rheb is an essential activator of mTORC1 signaling (Yang et al., 2017), and mTORC1 promotes lipogenesis (Kucejova et al., 2016; Yang et al., 2017); inactivating mTORC1 prevents the development of fatty liver induced by high-fat and -cholesterol diet (Peterson et al., 2011). Consistent with the reported role of mTORC1 in promoting lipid synthesis, we found that *Rheb* KO decreases mTORC1 activity (Figure 2A, Supplementary Figure S2A) and the expression of lipogenic genes such as *Srebp1-c* and *Fasn* (Figure 2B, Supplementary Figure S2B, C). However, genes related to fatty acid oxidation, such as *Cpt1α*, *Acad1*, and *Mcad* (Zou et al., 2014) were not dramatically altered (Figure 2C).

To further examine the accumulated lipids in hepatic *Rheb* KO mice, we performed lipidomic analysis on the *Rheb* KO liver and compared the results with that of the wild-type (WT) liver. By measuring the levels of 2,140 metabolites of the lipid metabolism pathway curated by Kyoto Encyclopedia of Genes and Genomes (KEGG) (Horton et al., 2002), we found that triglyceride (TAG), fatty acid (FA), and phosphatidate (PA) were increased in the *Rheb* KO liver; besides, phosphatidylcholine (PC), phosphatidylethanolamine (PE), phosphatidylinositol (PI), and diacylglycerol (DAG) was trended lower in the *Rheb* KO liver (Figure 2D, left panel, Supplementary Figure S3). Particularly, multiple forms of triglycerides were selectively increased by *Rheb* KO (Figure 2D, right panel). Biochemical assay confirmed that the triglyceride level was significantly increased in the liver of *Rheb* KO mice, but decreased in the serum (Figure 2E). Moreover, total cholesterol contents were reduced in both the liver and serum of *Rheb* KO (Figure 2F, Supplementary Figure S4A), suggesting that *Rheb* KO has a profound effect on the synthesis of hepatic cholesterol. Also, the serum level of fatty acids was not dramatically altered in the steady state by *Rheb* KO (Supplementary Figure S4B). Therefore, the increased triglyceride accounts for the hepatosteatosis in the *Rheb* KO mouse.

Serum triglyceride is mainly derived from hepatocytes in the form of very-low-density lipoprotein (VLDL) particles containing a hydrophobic core of triglyceride and an apolipoprotein B (ApoB) molecule (Ye et al., 2009). *Rheb* KO does not alter the expression of genes related to VLDL maturation (Supplementary Figure S2D, E). The decreased serum triglyceride suggests impairment in lipid secretion. To substantiate the notion that *Rheb* KO impairs hepatic triglyceride secretion to the serum, we assessed ApoB protein levels in the serum of *Rheb* KO and found a significant reduction in the serum (Figures 2G,H), which is consistent with reduced lipid secretion. To further validate the impairment in lipid secretion, we examined the VLDL content in the serum of *Rheb* KO and control mice with or without the injection of Triton WR-1339. Triton WR-1339 inhibits various forms of lipase and therefore, blunts the effect of serum VLDL breakdown. We found that the VLDL content was low and comparable in normal control and *Rheb* KO mice under the steady state (Figure 2I). Triton WR-1339 significantly elevated the VLDL content in both control and *Rheb* KO mice, but the serum from control mice was paler and more turbid than that of the *Rheb* KO mouse (Supplementary Figure S2F), and VLDL in the serum of the *Rheb* KO mouse was significantly lower than that of the control (Figure 2I), suggesting that the amount of VLDL secreted from the liver was reduced. All these results indicate that *Rheb* controls hepatic triglyceride secretion, and the deficiency in triglyceride secretion caused by *Rheb* KO is a fundamental error behind hepatosteatosis in the *Rheb* KO mouse.

### Exogeneous ATP Ameliorates Hepatosteatosis in *Rheb* KO Mice

Triglyceride secretion is a dynamic energy-consuming process that is linked to mitochondrial energy production (Hasuzawa et al., 2021). We have showed that Rheb regulates mitochondrial energy production through pyruvate dehydrogenase (PDH) and *Rheb* KO in hepatocytes reduces hepatic ATP production (Yang et al., 2021). Therefore, we reasoned that *Rheb* could regulate triglyceride secretion through PDH-controlled ATP production and *Rheb* KO–induced hepatosteatosis could be causally related to hepatic PDH inactivation and decreased energy production (Yang et al., 2021). As a first step toward examining the role of PDH in Rheb-regulated lipid secretion, we first tried to apply the only PDH activator available—dichloroacetic acid (DCA) that inhibits PDH kinases (PDKs)—to bring down the level of PDH phosphorylation in *Rheb* KO cells, but failed (Supplementary Figures S5A, B). Therefore, it is technically impossible to restore PDH activity in the liver of *Rheb* KO mice through pharmacological manipulation.

**FIGURE 3:**
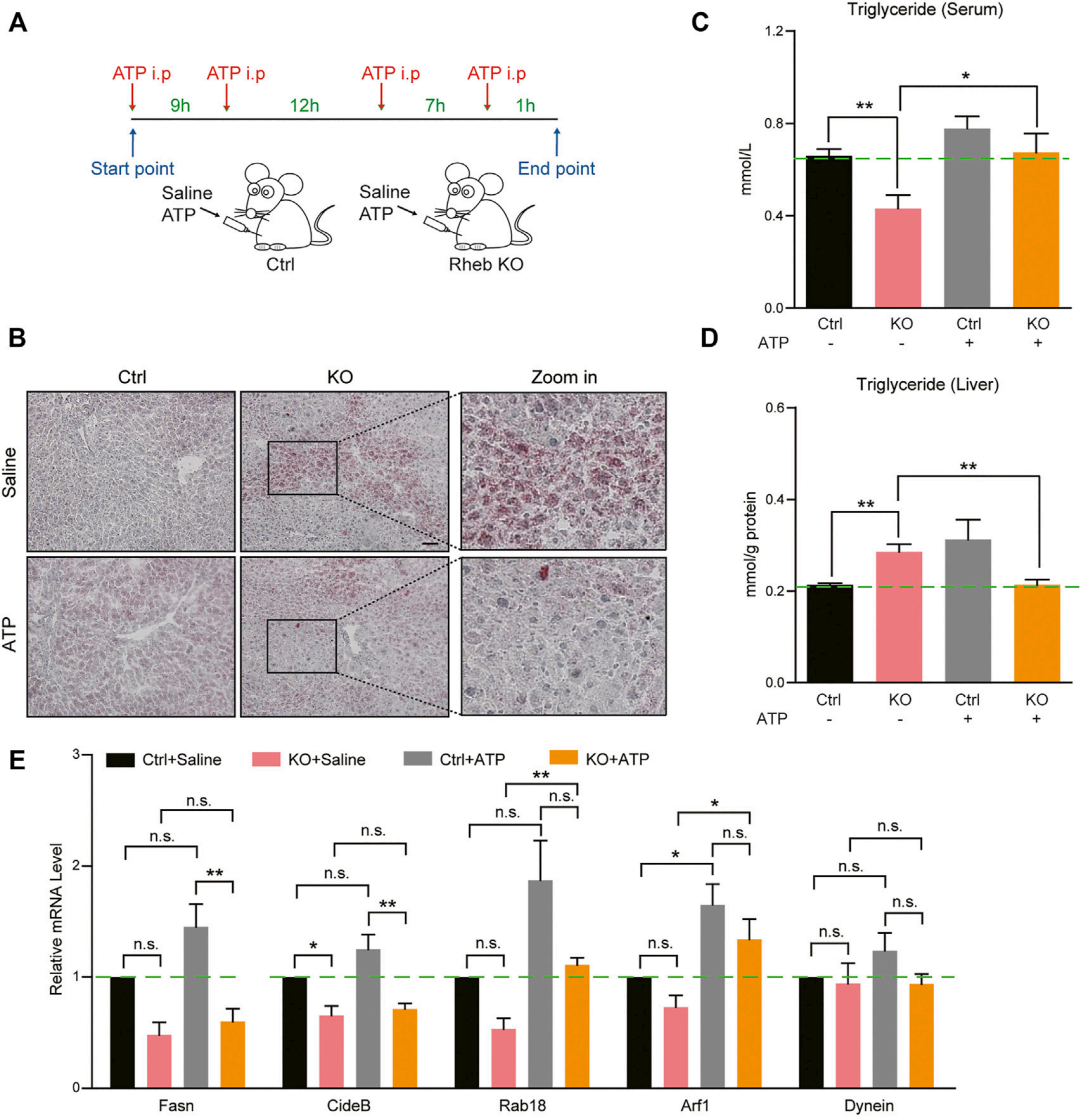
ATP supplements ameliorate hepatosteatosis in *Rheb* KO mice. **(A)** A schematic illustration that contains control and *Rheb* KO mice injected with saline or ATP (dosage: 125 μg/g) four times. **(B)** Images of Oil Red O staining showing the decreasing of lipid droplets in the liver of *Rheb* KO mice by ATP treatment. **(C-D)** Biochemical assays showing the decreased triglyceride level in the liver and increased in the serum in *Rheb* KO mice by ATP treatment. **(E)** mRNA levels of genes related to lipid droplet secretion in the liver of *Rheb* KO mice by ATP treatment. Data represent mean ± SEM. *p < 0.05, **p < 0.01. n.s, no statistical significance.

Alternatively, we injected ATP molecules in *Rheb* KO mice to restore hepatic ATP levels (Figure 3A). Oil red O staining showed that ATP supplement partly ameliorated the hepatosteatosis in the liver of *Rheb* KO mice (Figure 3B). Biochemical assays confirmed that additional ATP decreased the triglyceride level in the liver and increased the triglyceride level in the serum of *Rheb* KO mice (Figures 3C,D). To demonstrate that this effect on hepatic triglyceride secretion is a direct result of exogenous ATP acting on hepatocytes, we set up a hepatocyte culture of mouse livers and tested the effect of ATP treatment on triglyceride secretion. The results show that addition of ATP decreased the triglyceride (TAG) level in *Rheb* KO hepatocytes (Supplementary Figure S6A). In addition, we show that alpha-ketoglutarate (α-KG, the substrate of the TCA cycle) treatment also decreased triglyceride contents in the liver and increased the triglyceride level in the serum of *Rheb* KO (Supplementary Figure S6B, C). These findings support the notion that Rheb-regulated mitochondrial energy metabolism plays a role in regulating hepatic triglyceride secretion. In addition, we found that ATP treatment increased the expressions of genes related to lipid droplet secretion, including *Rab18* and *Arf1* in the liver of the *Rheb* KO mouse (Figure 3E). The increased expression of Rab18 and Arf1 could contribute to the hepatic triglyceride secretion.

### Antagonistic Effect of the *Rheb* Transgene Against Diet-Induced Hepatosteatosis

Based on findings with the *Rheb* KO mouse, we reasoned that increased Rheb activity would have a prophylactic effect against hepatosteatosis. To test this hypothesis, we generated *Rheb* liver-specific transgenic mice by knocking in *Rheb S16H* into the *Rosa26* locus in hepatocytes with albumin-cre. In this mouse model, *Rheb S16H* constitutively activates mTORC1 in the liver but did not affect the systematic or liver development under normal diet (Supplementary Figure S7A–D). Also, lipogenesis genes (*Srebp1-c, ACC1, and Fasn*) were increased in the *Rheb S16H* transgenic liver (Supplementary Figure S7E). Importantly, the ATP level in the *Rheb* transgenic liver was increased (Supplementary Figure S7F).

**FIGURE 4:**
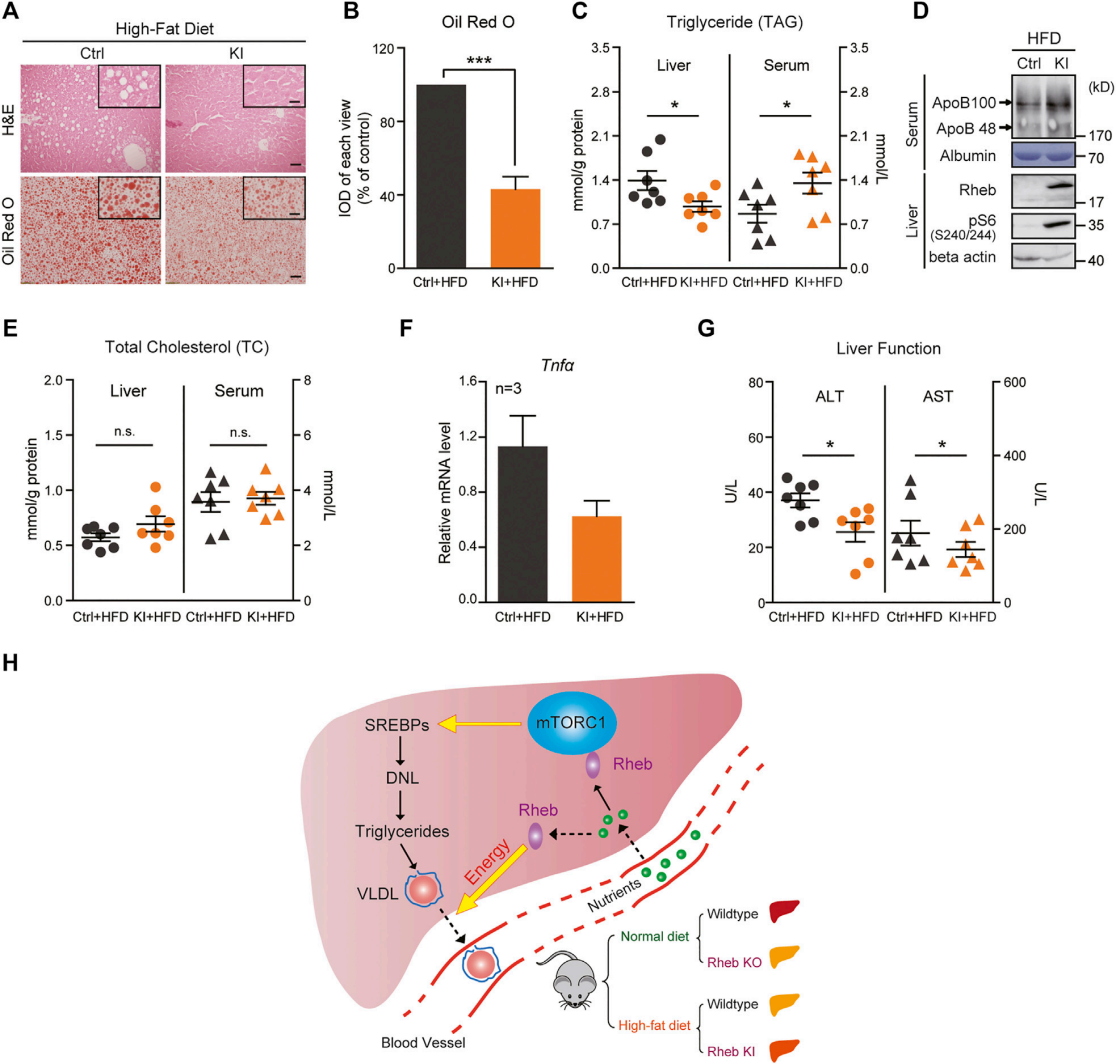
Overexpression of Rheb ameliorates hepatosteatosis and rescues liver function. **(A)** H and E (upper panel) and Oil Red O (lower panel) staining showing the *Rheb S16H* transgene reducing lipid levels in the liver of mice on HFD. Ctrl mice, *Rheb* K/+, or *Rheb* K/K. *Rheb* transgene mice, *Rheb S16H* K/+; alb-Cre. Scale bar, 50 and 10 μm (zoom in). **(B)** Quantification of Oil Red O staining in Figure 4A. **(C)** Diagram showing decreased liver triglyceride and increased serum triglyceride levels in *Rheb S16H* mice on HFD (*n* = 7). **(D)** Increased ApoB proteins in the serum of *Rheb S16H* transgenic mice on HFD. **(E)** Diagram showing the total cholesterol contents in the liver and serum of *Rheb S16H* mice on HFD (*n* = 7). **(F)** Decreased mRNA levels of inflammatory cytokines in *Rheb S16H* transgenic mice (HFD, *n* = 3). **(G)** Decreased ALT and AST levels in the serum of *Rheb S16H* transgenic mice on HFD (*n* = 7). **(H)** A model summarizing that *Rheb* controls hepatic triglyceride secretion by regulating energy production. Elevation of *Rheb* expression resists HFD-induced fatty livers. Data represent mean ± SEM. *p < 0.05 and ***p < 0.001. n.s, no statistical significance.

Then, we tested the efficacy of the *Rheb* transgene against high-fat diet (HFD)–induced steatosis. Remarkably, we found that *Rheb* transgene dramatically decreased the ballooning-like degeneration and lipid accumulation in the liver, which is normally associated with HFD-induced hepatosteatosis (Figures 4A,B, Supplementary Figures S8A–E). Biochemical measurements showed that the liver triglyceride level of the *Rheb* transgenic mouse was lower than that of the controls. Conversely, the serum triglyceride level of the *Rheb* transgenic mouse was higher than that of the controls (Figure 4C). Also, the ApoB level was increased in the serum of the *Rheb* transgenic mouse (Figure 4D, Supplementary Figure S8F). The total cholesterol contents in the *Rheb* transgene liver does not decrease or increase in the serum (Figure 4E), suggesting that the *Rheb*’s effect of anti-hepatosteatosis was selectively on triglyceride. Consistent with a protective effect of the *Rheb* transgene on HFD-induced liver damage, in the *Rheb* transgenic mice, the liver damage, as indicated by inflammatory cytokines (*Tnfα*) and the release of hepatic enzymes (ALT and AST), was reduced (Figures 4F,G). All these results indicate that the *Rheb* transgene was effective in antagonizing diet-induced hepatosteatosis and preserving hepatic function.

## Discussion

Our study identifies a novel function of Rheb in the regulation of hepatic lipid homeostasis, which is to regulate triglyceride secretion. We showed that genetic deletion of *Rheb* causes robust, spontaneous development of hepatosteatosis and expression of the *Rheb* transgene ameliorates lipid accumulation induced by HFD (Figure 4H). The development of hepatosteatosis in the *Rheb* KO mouse is unpredicted from the role of mTORC1 in activating *de novo* lipid synthesis and inhibiting autophagy (Peterson et al., 2011; He et al., 2020). Previous studies emphasize the role of Rheb in activating mTORC1 in mammalian cells. Activated mTORC1 promotes *de novo* lipid synthesis through transcriptional and post-transcriptional mechanisms (Lee et al., 2017). Therefore, it is anticipated that reducing mTORC1 activity would alleviate hepatosteatosis and be therapeutically beneficial to a fatty liver (Peterson et al., 2011). Our study adds support to the notion that simply suppressing hepatic mTORC1 activity does not offer a therapeutic value in managing hepatosteatosis; inhibiting mTORC1 by administering the mTORC1 inhibitor rapamycin does not reduce HFD-induced lipid accumulation in the liver (Kenerson et al., 2011). On the contrary, we found that the *Rheb* transgene that activates mTORC1 prevented diet-induced chronic ailments and preserved liver functions, without significant effects on *de novo* lipid synthesis. This finding suggests that targeted enhancement of Rheb expression in hepatocytes could be of a therapeutic value for managing steatosis.

We found that the hepatosteatosis in the *Rheb* KO mouse is attributable to selective triglyceride accumulation in the liver. Results further suggest that triglyceride accumulation is due to impairment in VLDL-mediated triglyceride secretion because the accumulation of triglyceride in the liver is accompanied by the reduced serum triglyceride level and VLDL. How does Rheb regulate hepatic triglyceride secretion? Previous studies of *TSC1* and *Raptor* KO mouse models suggest that mTORC1 promotes hepatic lipid secretion through phosphatidylcholine (PC) synthesis (Quinn et al., 2017). In a *Raptor* KO liver, the amount of PC produced by hepatocytes is reduced by about 70% and the rate-limiting enzyme for PC synthesis phosphocholine cytidylyltransferase *α* (CCTα) is also significantly reduced (Quinn et al., 2017). We found that a hepatic *Rheb* KO mouse shows a tendency of a lower PC level (Supplementary Figure S9A) but does not reach the level of statistical significance. Also, CCTα was not altered by *Rheb* KO (Supplementary Figure S9B, C). Both PC and CCTα does not change in the Rheb transgene mice liver with normal and high-fat diet (Supplementary Figures S9D–I). All these results suggest that triglyceride accumulation caused by *Rheb* KO may not be mediated by the mTORC1-PC axis. In the hepatic TSC1 deletion model, the effect of TSC1 on hepatic mTORC1 and the triglyceride content is mainly manifested under fasted condition (Cornu et al., 2014) and appears to be mediated by activated expression of the transcriptional activator PGC1a that promotes lipid oxidation and the stress hormone FGF21 (Bookout et al., 2013). We found that serum FGF21 was lower in the *Rheb* KO mouse (Supplementary Figure S10A). These results suggest that the mTORC1-FGF21 axis could be involved in Rheb-regulated hepatic triglyceride secretion. On the other hand, we found that the serum FGF21 level of the *Rheb* transgene mice does not change (Supplementary Figure S10B-C).

Our studies of Rheb KO and KI models suggest that Rheb-regulated mitochondrial energy metabolism and ATP production could play a role in Rheb-regulated lipid secretion. The secretion of lipid is an energy-consuming process (Rector et al., 2010; van Zutphen et al., 2016). *Rheb* KO impairs mitochondrial PDH activity of hepatocytes and their ATP production, whereas the *Rheb* transgene activates PDH activity and enhances ATP production of hepatocytes (Yang et al., 2021). We also found that exogenous ATP supplement could partly ameliorate the steatosis in the liver of the *Rheb* KO mouse, which links hepatic energy-deficit to impaired lipid secretion. Of note, our finding that Rheb plays a role in haptic triglyceride secretion is contradictory to the result obtained by injecting the adenovirus expressing the *Rheb* transgene to mice fed with normal diet (Uno et al., 2015). The *Rheb* transgene activates mTORC1 in the liver, and serum triglyceride content is increased, but hepatic triglyceride secretion does not seem to be altered in the mice fed with normal chow diet. Instead, the increase in serum triglyceride is shown to be a result of decreased expression of the adipose lipoprotein lipase (that inhibits triglyceride breakdown) in white adipose tissue. A notable difference is that our study is based on the Cre-dependent expression of the constitutively active *Rheb (S16H)* that is resistant to TSC inhibition (Yan et al., 2006), and this active *Rheb* transgene counteracts hepatosteatosis induced by a high-fat diet.

In conclusion, we demonstrate that genetic deletion of *Rheb* causes rapid and spontaneous steatosis in the liver. Reciprocally, the *Rheb* transgene remarkably reduces diet-induced hepatosteatosis. The altered lipid homeostasis in *Rheb* KO and KI mouse models could be attributable to both mTORC1-independent, PDH-regulated ATP production and mTORC1-depdenent lipid secretion involving FGF21. Our findings highlight an underappreciated role of Rheb-regulated energy production in hepatic lipid secretion, with potential therapeutic implication.

**TABLE 1:** qPCR primers in this study.

Primers	Forward (5′-3′)	Reverse (5′-3′)
Genes		
*Srebp1-c*	GGA​GCC​ATG​GAT​TGC​ACA​TT	GCT​TCC​AGA​GAG​GAG​GCC​AG
*Fasn*	GGA​GGT​GGT​GAT​AGC​CGG​TAT	TGG​GTA​ATC​CAT​AGA​GCC​CAG
*Acl*	GAA​GCT​GAC​CTT​GCT​GAA​CC	CTG​CCT​CCA​ATG​ATG​AGG​AT
*Pparα*	TGT​TTG​TGG​CTG​CTA​TAA​TTT​GC	GCA​ACT​TCT​CAA​TGT​AGC​CTA​TGT​TT
*Acsl1*	TGC​CAG​AGC​TGA​TTG​ACA​TTC	GGC​ATA​CCA​GAA​GGT​GGT​GAG
*Cpt1α*	GGA​GAG​AAT​TTC​ATC​CAC​TTC​CA	CTT​CCC​AAA​GCG​GTG​TGA​GT
*Mcad*	TTT​CGA​AGA​CGT​CAG​AGT​GC	TGC​GAC​TGT​AGG​TCT​GGT​TC
*Acad1*	TCT​TTT​CCT​CGG​AGC​ATG​ACA	GAC​CTC​TCT​ACT​CAC​TTC​TCC​AG
*Mlycd*	GCA​CGT​CCG​GGA​AAT​GAA​C	GCC​TCA​CAC​TCG​CTG​ATC​TT
*Tnfα*	CCC​TCA​CAC​TCA​GAT​CAT​CTT​CT	GCT​ACG​ACG​TGG​GCT​ACA​G
*CideB*	CAA​TGG​CCT​GCT​AAG​GTC​AGT	GAT​CAC​AGA​CAC​GGA​AGG​GTC
*Rab18*	TTT​GCA​CGC​AAG​CAT​TCT​ATG​T	TTG​TTC​TGG​TTC​TCA​CTT​TCC​C
*Arf1*	TGG​GCG​AAA​TTG​TGA​CCA​CC	TCC​ACT​ACG​AAG​ATC​AAG​CCT
*Dynein*	AAG​CAC​CTG​CGT​AAG​CTG​G	GCG​GGT​CTG​ACA​GGA​ACT​TG

## Data Availability

All relevant data are available from the corresponding authors, upon reasonable request.

## References

[B1] Baiceanu A. Mesdom P. Lagouge M. Foufelle F. (2016). Endoplasmic Reticulum Proteostasis in Hepatic Steatosis. Nat. Rev. Endocrinol. 12, 710–722. 10.1038/nrendo.2016.124 27516341

[B2] Bechmann L. P. Hannivoort R. A. Gerken G. Hotamisligil G. S. Trauner M. Canbay A. (2012). The Interaction of Hepatic Lipid and Glucose Metabolism in Liver Diseases. J. Hepatol. 56, 952–964. 10.1016/j.jhep.2011.08.025 22173168

[B3] Bookout A. L. de Groot M. H. M. Owen B. M. Lee S. Gautron L. Lawrence H. L. (2013). FGF21 Regulates Metabolism and Circadian Behavior by Acting on the Nervous System. Nat. Med. 19, 1147–1152. 10.1038/nm.3249 23933984 PMC3769420

[B4] Borensztajn J. Rone M. S. Kotlar T. J. (1976). The Inhibition *In Vivo* of Lipoprotein Lipase (Clearing-factor Lipase) Activity by Triton WR-1339. Biochem. J. 156, 539–543. 10.1042/bj1560539 949335 PMC1163786

[B5] Cohen J. C. Horton J. D. Hobbs H. H. (2011). Human Fatty Liver Disease: Old Questions and New Insights. Science 332, 1519–1523. 10.1126/science.1204265 21700865 PMC3229276

[B6] Cornu M. Oppliger W. Albert V. Robitaille A. M. Trapani F. Quagliata L. (2014). Hepatic mTORC1 Controls Locomotor Activity, Body Temperature, and Lipid Metabolism through FGF21. Proc. Natl. Acad. Sci. 111, 11592–11599. 10.1073/pnas.1412047111 25082895 PMC4136616

[B7] Crescenzo R. Bianco F. Falcone I. Coppola P. Liverini G. Iossa S. (2013). Increased Hepatic De Novo Lipogenesis and Mitochondrial Efficiency in a Model of Obesity Induced by Diets Rich in Fructose. Eur. J. Nutr. 52, 537–545. 10.1007/s00394-012-0356-y 22543624

[B8] Delgoffe G. M. Pollizzi K. N. Waickman A. T. Heikamp E. Meyers D. J. Horton M. R. (2011). The Kinase mTOR Regulates the Differentiation of Helper T Cells through the Selective Activation of Signaling by mTORC1 and mTORC2. Nat. Immunol. 12, 295–303. 10.1038/ni.2005 21358638 PMC3077821

[B9] Hasuzawa N. Tatsushima K. Wang L. Kabashima M. Tokubuchi R. Nagayama A. (2021). Clodronate, an Inhibitor of the Vesicular Nucleotide Transporter, Ameliorates Steatohepatitis and Acute Liver Injury. Sci. Rep. 11, 5192. 10.1038/s41598-021-83144-w 33664289 PMC7933178

[B10] He A. Chen X. Tan M. Chen Y. Lu D. Zhang X. (2020). Acetyl-CoA Derived from Hepatic Peroxisomal β-Oxidation Inhibits Autophagy and Promotes Steatosis via mTORC1 Activation. Mol. Cel. 79, 30–42. 10.1016/j.molcel.2020.05.007 PMC7335356 32473093

[B11] Horton J. D. Goldstein J. L. Brown M. S. (2002). SREBPs: Activators of the Complete Program of Cholesterol and Fatty Acid Synthesis in the Liver. J. Clin. Invest. 109, 1125–1131. 10.1172/jci0215593 11994399 PMC150968

[B12] Jia L. Liao M. Mou A. Zheng Q. Yang W. Yu Z. (2021). Rheb-regulated Mitochondrial Pyruvate Metabolism of Schwann Cells Linked to Axon Stability. Developmental Cel. 56, 2980–2994. e2986. 10.1016/j.devcel.2021.09.013 34619097

[B13] Kenerson H. L. Yeh M. M. Yeung R. S. (2011). Tuberous Sclerosis Complex-1 Deficiency Attenuates Diet-Induced Hepatic Lipid Accumulation. PloS one 6, e18075. 10.1371/journal.pone.0018075 21479224 PMC3066210

[B14] Kucejova B. Duarte J. Satapati S. Fu X. Ilkayeva O. Newgard C. B. (2016). Hepatic mTORC1 Opposes Impaired Insulin Action to Control Mitochondrial Metabolism in Obesity. Cel Rep. 16, 508–519. 10.1016/j.celrep.2016.06.006 PMC4951107 27346353

[B15] Lee G. Zheng Y. Cho S. Jang C. England C. Dempsey J. M. (2017). Post-transcriptional Regulation of De Novo Lipogenesis by mTORC1-S6k1-SRPK2 Signaling. Cell 171, 1545–1558. 10.1016/j.cell.2017.10.037 29153836 PMC5920692

[B16] Maillo C. Martín J. Sebastián D. Hernández-Alvarez M. García-Rocha M. Reina O. (2017). Circadian- and UPR-dependent Control of CPEB4 Mediates a Translational Response to Counteract Hepatic Steatosis under ER Stress. Nat. Cel Biol 19, 94–105. 10.1038/ncb3461 28092655

[B17] Morral N. Edenberg H. J. Witting S. R. Altomonte J. Chu T. Brown M. (2007). Effects of Glucose Metabolism on the Regulation of Genes of Fatty Acid Synthesis and Triglyceride Secretion in the Liver. J. lipid Res. 48, 1499–1510. 10.1194/jlr.m700090-jlr200 17449907

[B18] Musso G. Gambino R. Cassader M. (2009). Recent Insights into Hepatic Lipid Metabolism in Non-alcoholic Fatty Liver Disease (NAFLD). Prog. lipid Res. 48, 1–26. 10.1016/j.plipres.2008.08.001 18824034

[B19] Peterson T. R. Sengupta S. S. Harris T. E. Carmack A. E. Kang S. A. Balderas E. (2011). mTOR Complex 1 Regulates Lipin 1 Localization to Control the SREBP Pathway. Cell 146, 408–420. 10.1016/j.cell.2011.06.034 21816276 PMC3336367

[B20] Quinn W. J. 3rd Wan M. Shewale S. V. Gelfer R. Rader D. J. Birnbaum M. J. (2017). mTORC1 Stimulates Phosphatidylcholine Synthesis to Promote Triglyceride Secretion. J. Clin. Invest. 127, 4207–4215. 10.1172/jci96036 29035283 PMC5663357

[B21] Rector R. S. Thyfault J. P. Uptergrove G. M. Morris E. M. Naples S. P. Borengasser S. J. (2010). Mitochondrial Dysfunction Precedes Insulin Resistance and Hepatic Steatosis and Contributes to the Natural History of Non-alcoholic Fatty Liver Disease in an Obese Rodent Model. J. Hepatol. 52, 727–736. 10.1016/j.jhep.2009.11.030 20347174 PMC3070177

[B22] Ribes G. Valette G. Loubatières-Mariani M.-M. (1979). Metabolic Effects of Sodium Dichloroacetate in normal and Diabetic Dogs. Diabetes 28, 852–857. 10.2337/diab.28.9.852 111983

[B23] Sakai N. Van Sweringen H. L. Quillin R. C. Schuster R. Blanchard J. Burns J. M. (2012). Interleukin-33 Is Hepatoprotective during Liver Ischemia/reperfusion in Mice. Hepatology 56, 1468–1478. 10.1002/hep.25768 22782692 PMC3465516

[B24] Saxton R. A. Sabatini D. M. (2017). mTOR Signaling in Growth, Metabolism, and Disease. Cell 169, 361–371. 10.1016/j.cell.2017.03.035 28388417

[B25] Seglen P. O. (1976). Chapter 4 Preparation of Isolated Rat Liver Cells. Methods Cel Biol 13, 29–83. 10.1016/s0091-679x(08)61797-5 177845

[B26] Stacpoole P. W. Harwood H. J. Jr. Varnado C. E. (1983). Regulation of Rat Liver Hydroxymethylglutaryl Coenzyme A Reductase by a New Class of Noncompetitive Inhibitors. Effects of Dichloroacetate and Related Carboxylic Acids on Enzyme Activity. J. Clin. Invest. 72, 1575–1585. 10.1172/jci111116 6630519 PMC370445

[B27] Tanaka S. Hikita H. Tatsumi T. Sakamori R. Nozaki Y. Sakane S. (2016). Rubicon Inhibits Autophagy and Accelerates Hepatocyte Apoptosis and Lipid Accumulation in Nonalcoholic Fatty Liver Disease in Mice. Hepatology 64, 1994–2014. 10.1002/hep.28820 27637015

[B28] Uno K. Yamada T. Ishigaki Y. Imai J. Hasegawa Y. Sawada S. (2015). A Hepatic Amino acid/mTOR/S6K-dependent Signalling Pathway Modulates Systemic Lipid Metabolism via Neuronal Signals. Nat. Commun. 6, 7940. 10.1038/ncomms8940 26268630 PMC4557134

[B29] van Zutphen T. Ciapaite J. Bloks V. W. Ackereley C. Gerding A. Jurdzinski A. (2016). Malnutrition-associated Liver Steatosis and ATP Depletion Is Caused by Peroxisomal and Mitochondrial Dysfunction. J. Hepatol. 65, 1198–1208. 10.1016/j.jhep.2016.05.046 27312946

[B30] Yan L. Findlay G. M. Jones R. Procter J. Cao Y. Lamb R. F. (2006). Hyperactivation of Mammalian Target of Rapamycin (mTOR) Signaling by a Gain-Of-Function Mutant of the Rheb GTPase. J. Biol. Chem. 281, 19793–19797. 10.1074/jbc.c600028200 16728407

[B31] Yang H. Jiang X. Li B. Yang H. J. Miller M. Yang A. (2017). Mechanisms of mTORC1 Activation by RHEB and Inhibition by PRAS40. Nature 552, 368–373. 10.1038/nature25023 29236692 PMC5750076

[B32] Yang W. Pang D. Chen M. Du C. Jia L. Wang L. (2021). Rheb Mediates Neuronal-Activity-Induced Mitochondrial Energetics through mTORC1-independent PDH Activation. Developmental Cel 56, 811–825. e816. 10.1016/j.devcel.2021.02.022 PMC9096910 33725483

[B33] Ye J. Li J. Z. Liu Y. Li X. Yang T. Ma X. (2009). Cideb, an ER- and Lipid Droplet-Associated Protein, Mediates VLDL Lipidation and Maturation by Interacting with Apolipoprotein B. Cel Metab. 9, 177–190. 10.1016/j.cmet.2008.12.013 19187774

[B34] Zhang M. Zhao Y. Li Z. Wang C. (2018). Pyruvate Dehydrogenase Kinase 4 Mediates Lipogenesis and Contributes to the Pathogenesis of Nonalcoholic Steatohepatitis. Biochem. biophysical Res. Commun. 495, 582–586. 10.1016/j.bbrc.2017.11.054 29128353

[B35] Zou J. Zhou L. Du X.-X. Ji Y. Xu J. Tian J. (2011). Rheb1 Is Required for mTORC1 and Myelination in Postnatal Brain Development. Developmental Cel 20, 97–108. 10.1016/j.devcel.2010.11.020 PMC3056331 21238928

[B36] Zou Y. Jiang W. Wang J. Li Z. Zhang J. Bu J. (2014). Oligodendrocyte Precursor Cell-Intrinsic Effect of Rheb1 Controls Differentiation and Mediates mTORC1-dependent Myelination in Brain. J. Neurosci. 34, 15764–15778. 10.1523/jneurosci.2267-14.2014 25411504 PMC4236405

