# Supplementary material for: Rheb Promotes Triglyceride Secretion and Ameliorates Diet-Induced Steatosis in the Liver
Source: Front Cell Dev Biol. 2022 Mar 16;10:808140. doi: 10.3389/fcell.2022.808140 (PMC8965806; doi:10.3389/fcell.2022.808140)
Supplement: Supplementary file 1 [file DataSheet1.doc]

**Supplementary Figures**

**Supplementary Figure 1. Basic information of *Rheb* liver KO mice.**


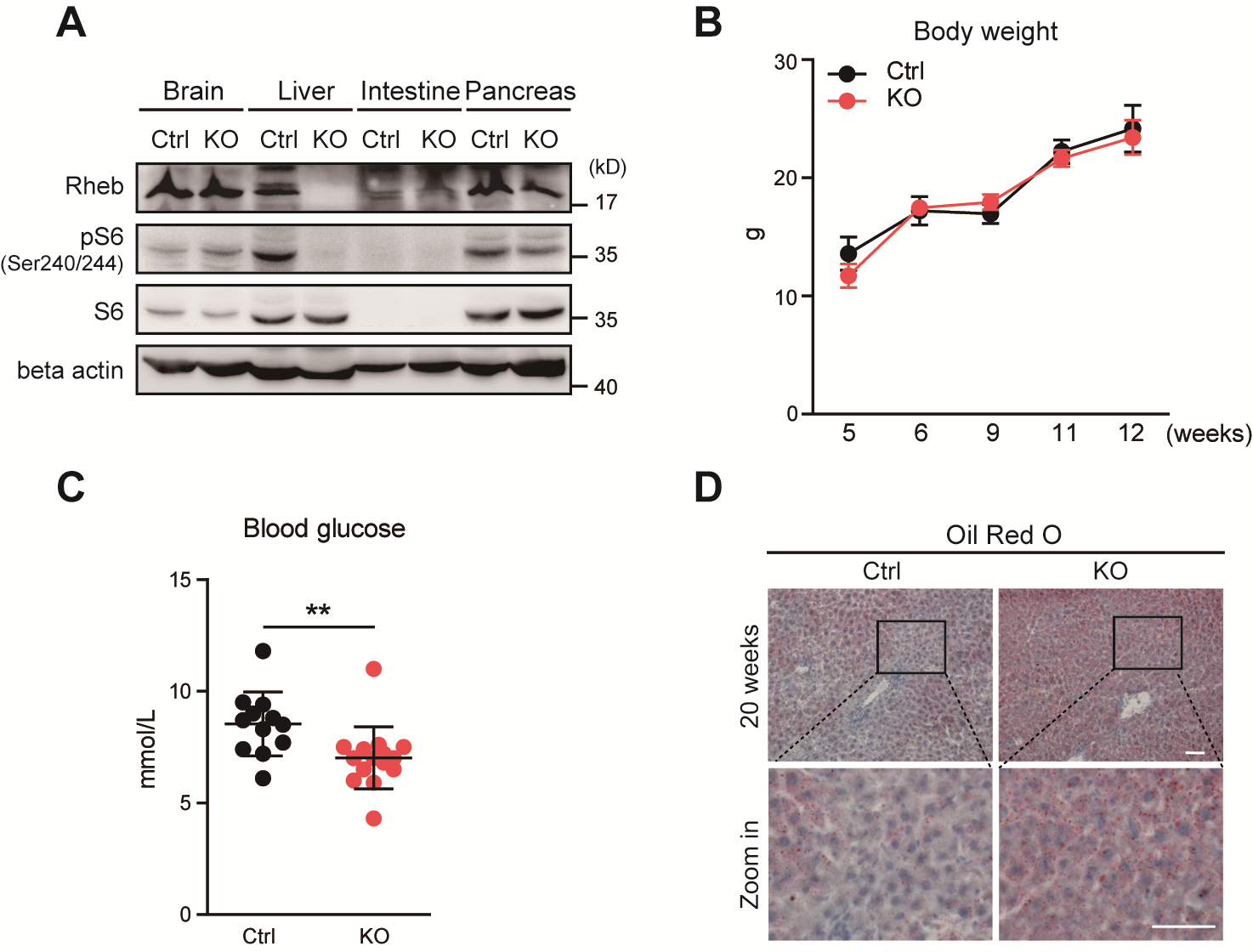


**(A)** Western blots showing the specificity of *Rheb* albumin-cre KO in the liver. **(B)** Body weight of control and *Rheb* KO mice from 5-week-old to 12-week-old. **(C)** The blood glucose level was slightly decreased in *Rheb* KO mice (Ctrl, n=12; KO, n=15). **(D)** Oil Red O staining showing increased lipid accumulation in the liver of *Rheb* KO mice (Age, 20 weeks). ***p*<0.01.

**Supplementary Figure 2. Lipid metabolism in *Rheb* KO liver.**


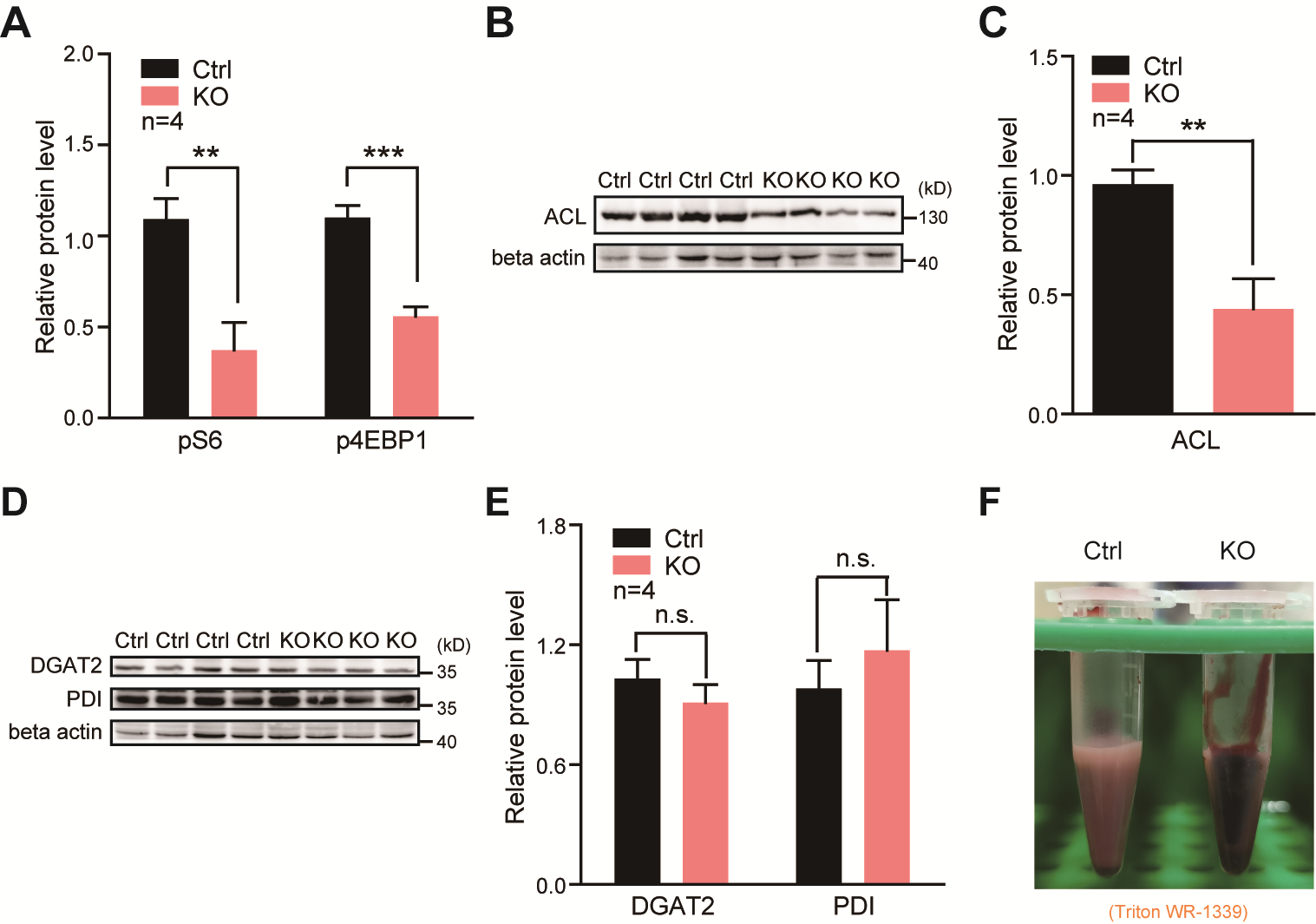


**(A)** The quantifications of mTORC1 activity in the liver of *Rheb* KO mice (n=4, normalized against beta actin). **(B-C)** Western blots **(B)** and quantifications **(C)** showing the decreased protein levels of lipogenic genes in the liver of *Rheb* KO (n=4, normalized against beta actin). **(D-E)** Western blots **(D)** and quantifications **(E)** showing the proteins related to VLDL maturation unchanged in *Rheb* KO (n=4, normalized against beta actin). **(F)** Images of serum samples from Ctrl and *Rheb* KO mice treated with Triton WR-1339 for 4 hours. Data represent mean ± SEM. ***p*<0.01, ****p*<0.001. n.s., no statistical significance.

**Supplementary Figure 3. Lipidomics in the liver of *Rheb* KO mice.**


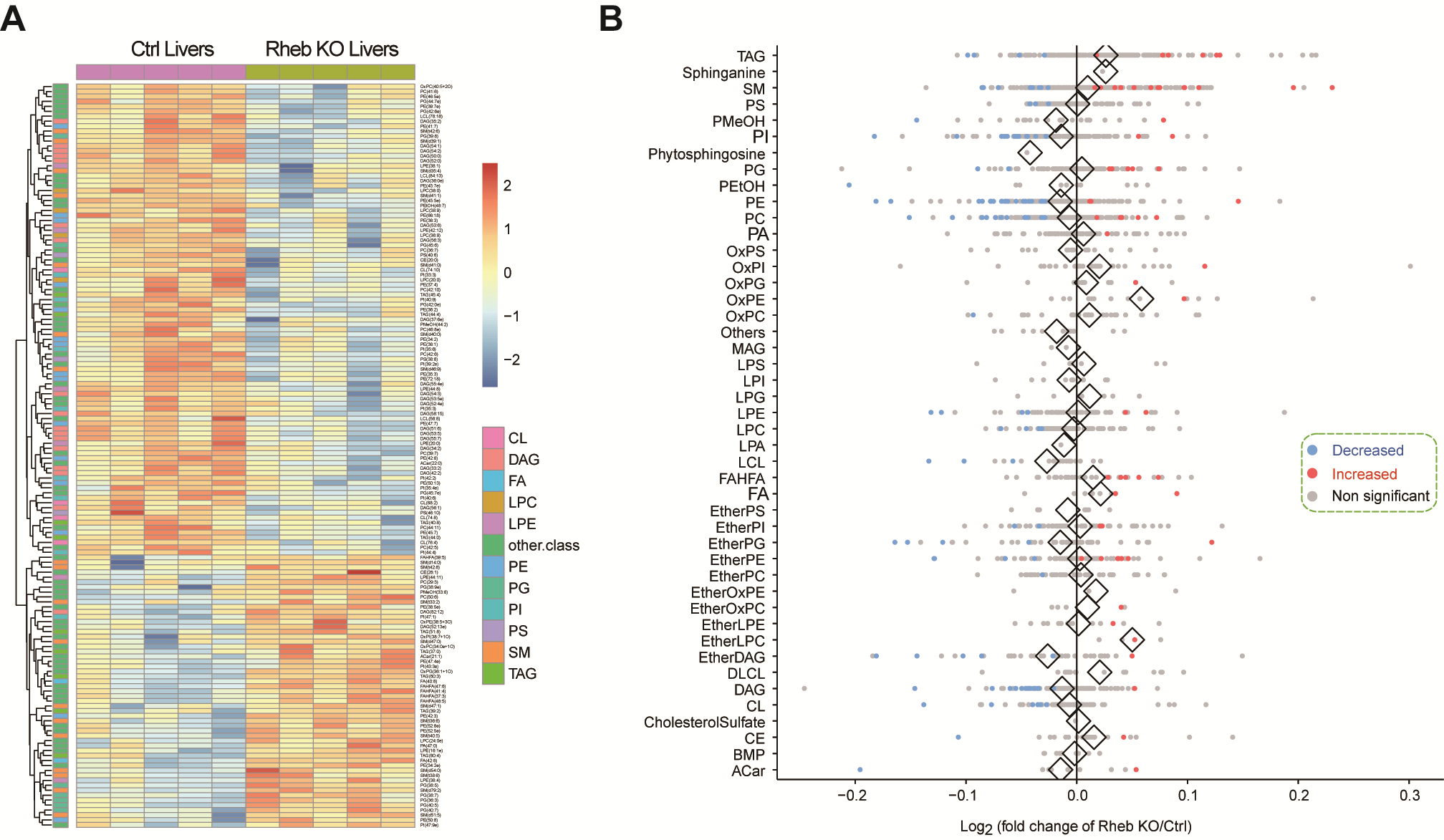


**(A)** Relative abundance (z-scores) of lipid metabolites in *Rheb* KO and control mice liver tissue, n=5. **(B)** Log fold changes of lipid metabolites in the liver of *Rheb* KO over control mice. Red and blue points represent significantly increased and decreased lipids of each lipid class in the liver of *Rheb* KO. Diamonds indicate the average log fold change of lipids in each lipid class.

**Supplementary Figure 4.** **Lipid metabolism and liver function in *Rheb* KO mice.**


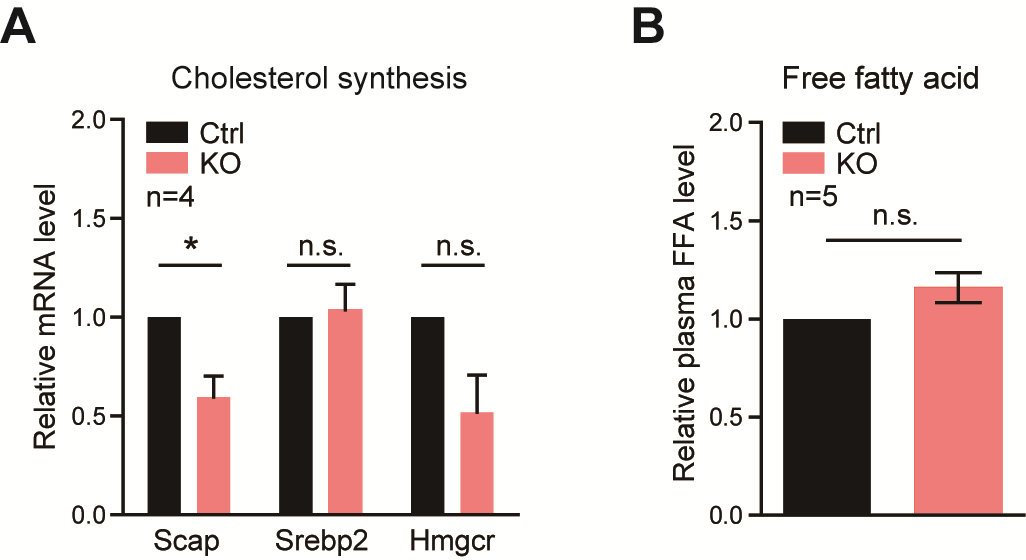


**(A)** Decreased mRNA levels of cholesterol synthesis genes in the liver of *Rheb* KO (n=4). **(B)** Unchanged free fatty acid levels in the serum of *Rheb* KO (n=5). Data represent mean ± SEM. **p*<0.05. n.s., no statistical significance.

**Supplementary Figure 5.** ***Rheb* knockdown increases PDH phosphorylation.**


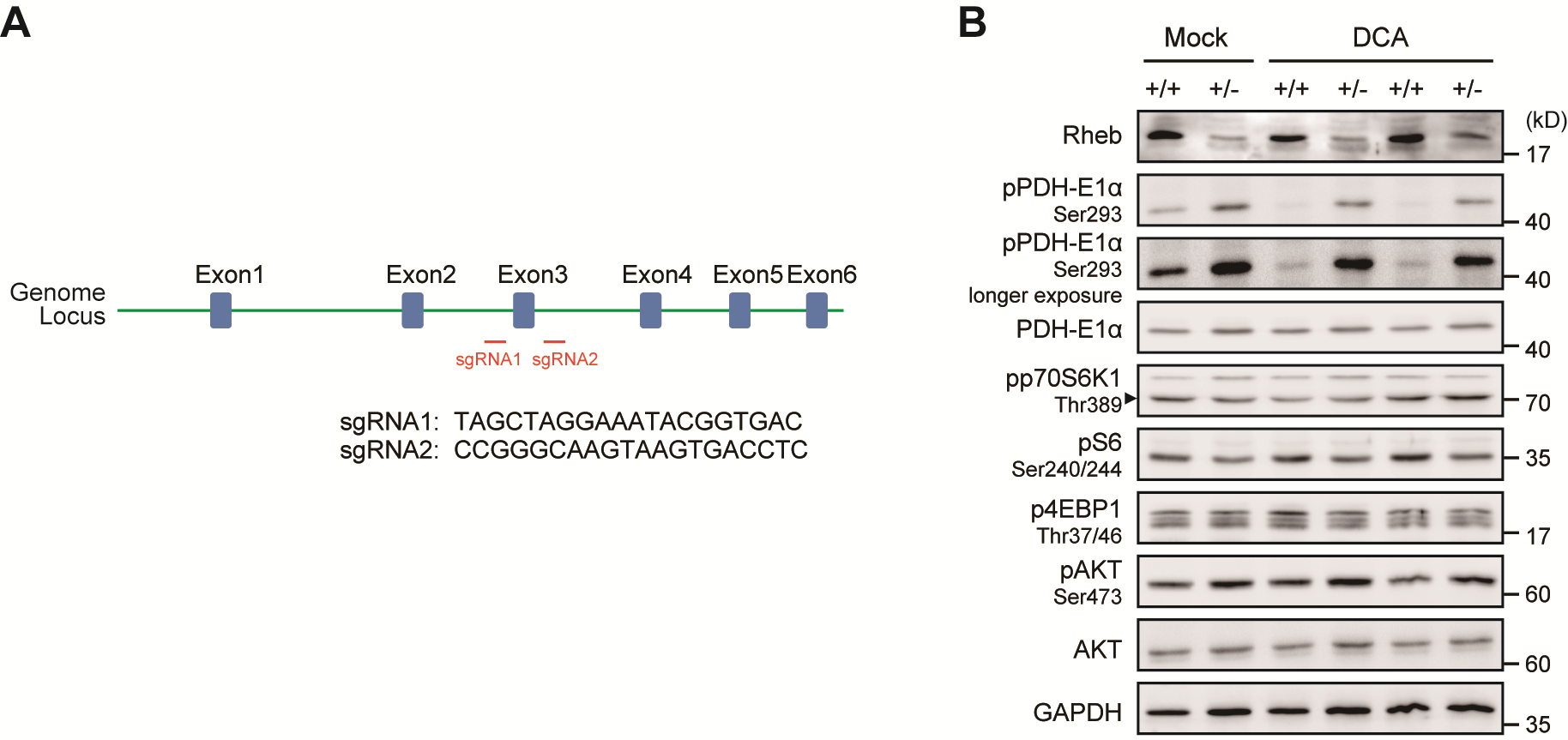


**(A)** A schematic model of *Rheb* gene knockout strategy by CRISPR/Cas9. The Cas9/sgRNAs target sites are indicated in red. **(B)** Western blots showing that *Rheb* knockdown in HEK293T cells increases PDH phosphorylation. DCA (dichloroacetic acid), the inhibitor of PDKs, 5 mM for overnight.

**Supplementary Figure 6. ATP supplements ameliorate hepatosteatosis in *Rheb* KO mice and hepatocytes.**


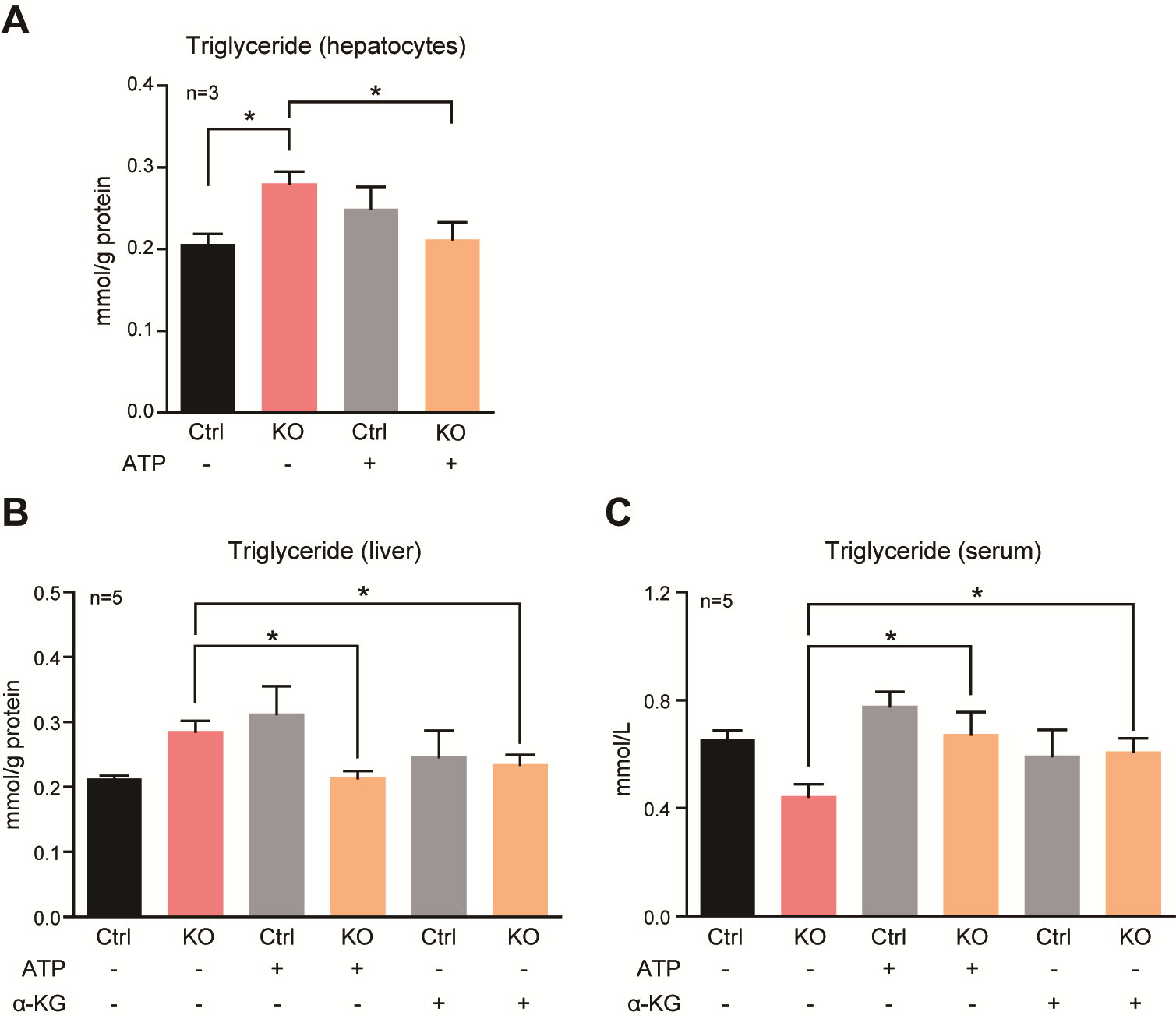


**(A)** Diagrams showing that triglycerides decreased in the hepatocyte of *Rheb* KO with ATP treatment. **(B)** Diagrams showing that triglyceride decreased in the liver of *Rheb* KO with ATP or α-KG treatment. **(C)** Diagrams showing that triglyceride increased in the serum of *Rheb* KO with ATP or α-KG treatment. Data represent mean ± SEM. **p*<0.05.

**Supplementary Figure 7. Basic information of *Rheb S16H* transgene mice (normal diet).**


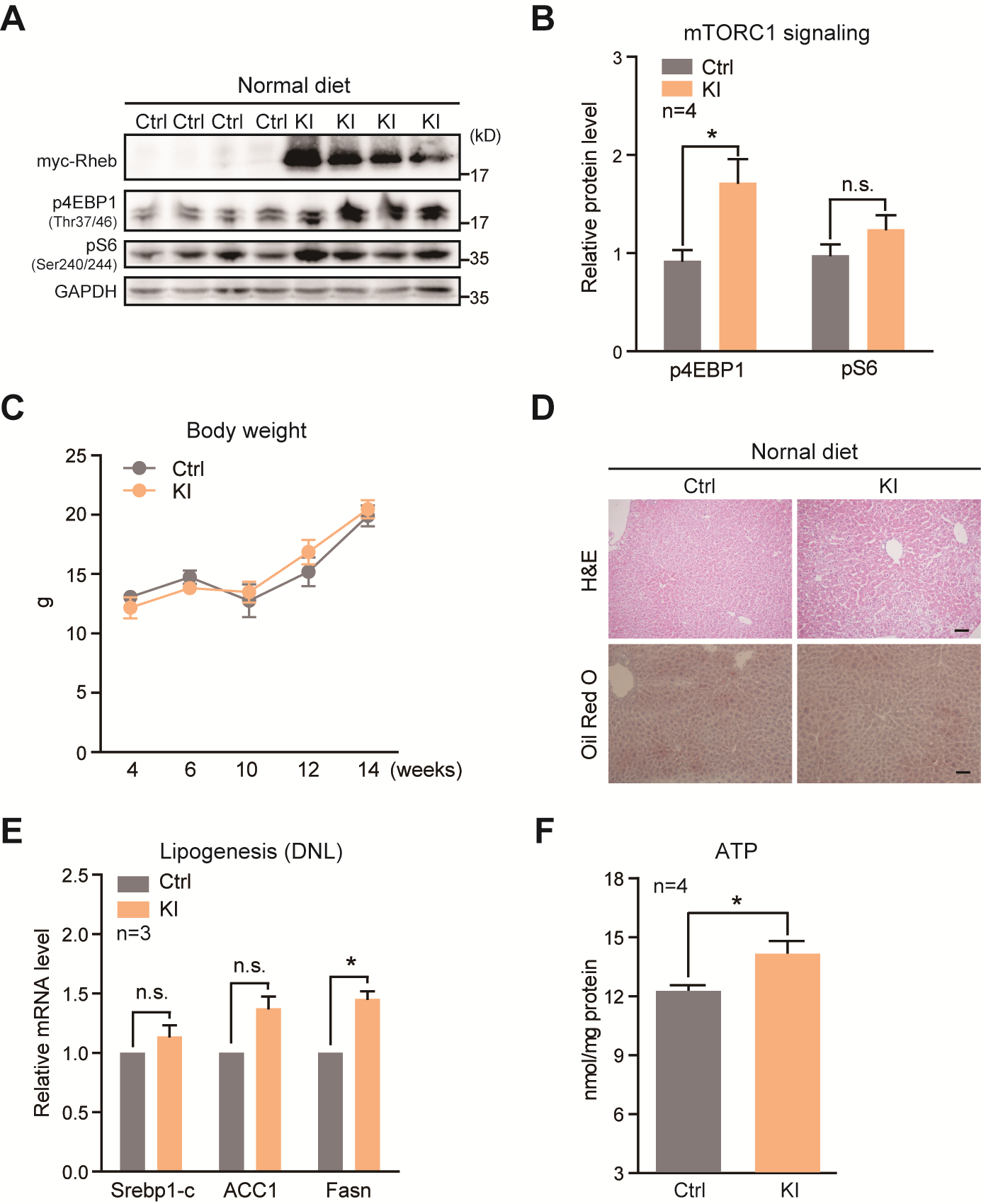


**(A-B)** Western blots **(A)** and quantifications **(B)** showing increased mTORC1 activity in the liver of *Rheb S16H* transgenic mice (normal diet, n=4, normalized against GAPDH). **(C)** Normal body weight of control and *Rheb S16H* transgenic mice from 4-week-old to 14-week-old (normal diet). **(D)** H&E (upper panel) and Oil red O (lower panel) staining showing comparable staining intensity of the liver from *Rheb S16H* transgenic and control mice on normal diet. **(E)** Quantitative RT–qPCR detection of lipogenic genes levels in the liver of *Rheb S16H* transgenic mice on normal diet (n=3). **(F)** ATP increased in the liver of *Rheb S16H* transgenic mice (normal diet, n=4). Data represent mean ± SEM. **p*<0.05. n.s., no statistical significance.

**Supplementary Figure 8. Basic information of *Rheb S16H* transgene mice (HFD).**


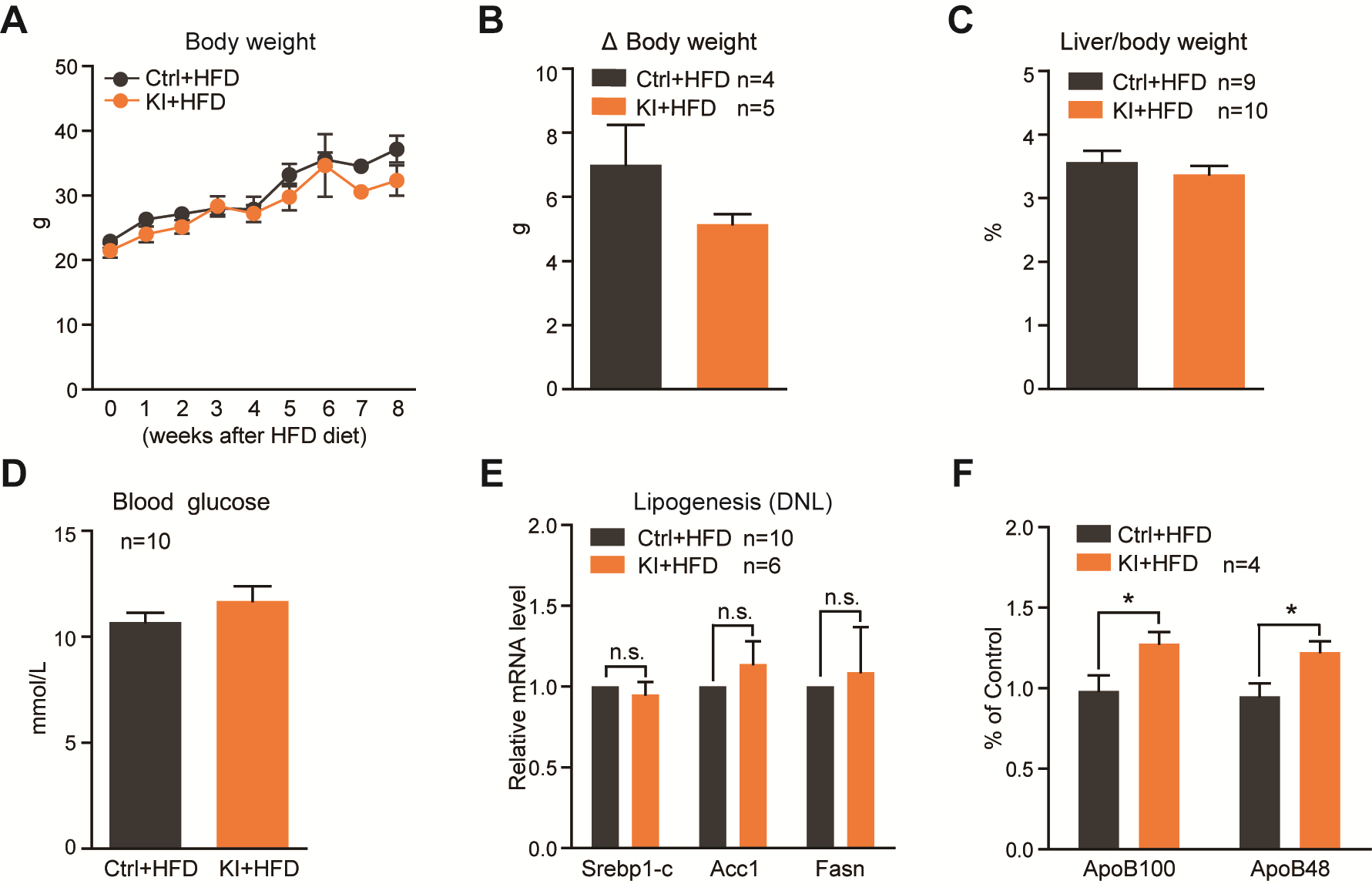


**(A)** Body weight of control and *Rheb S16H* transgenic mice by HFD for 8 weeks. **(B)** Altered body weight of control and *Rheb S16H* transgenic mice by HFD for 8 weeks. **(C)** The ratio of liver/body weight of control and *Rheb S16H* transgenic mice by HFD for 8 weeks. **(D)** The blood glucose of control and *Rheb S16H* transgenic mice by HFD for 8 weeks. **(E)** Quantitative RT–qPCR detection of lipogenic genes levels in the liver of *Rheb S16H* transgenic mice on high-fat diet. **(F)** Quantifications showing that ApoB levels were increased in the serum of *Rheb S16H* transgenic mice compared to Ctrl. Data represent mean ± SEM. **p*<0.05.

**Supplementary Figure 9. PC biosynthesis in the liver of *Rheb* KO and KI.**


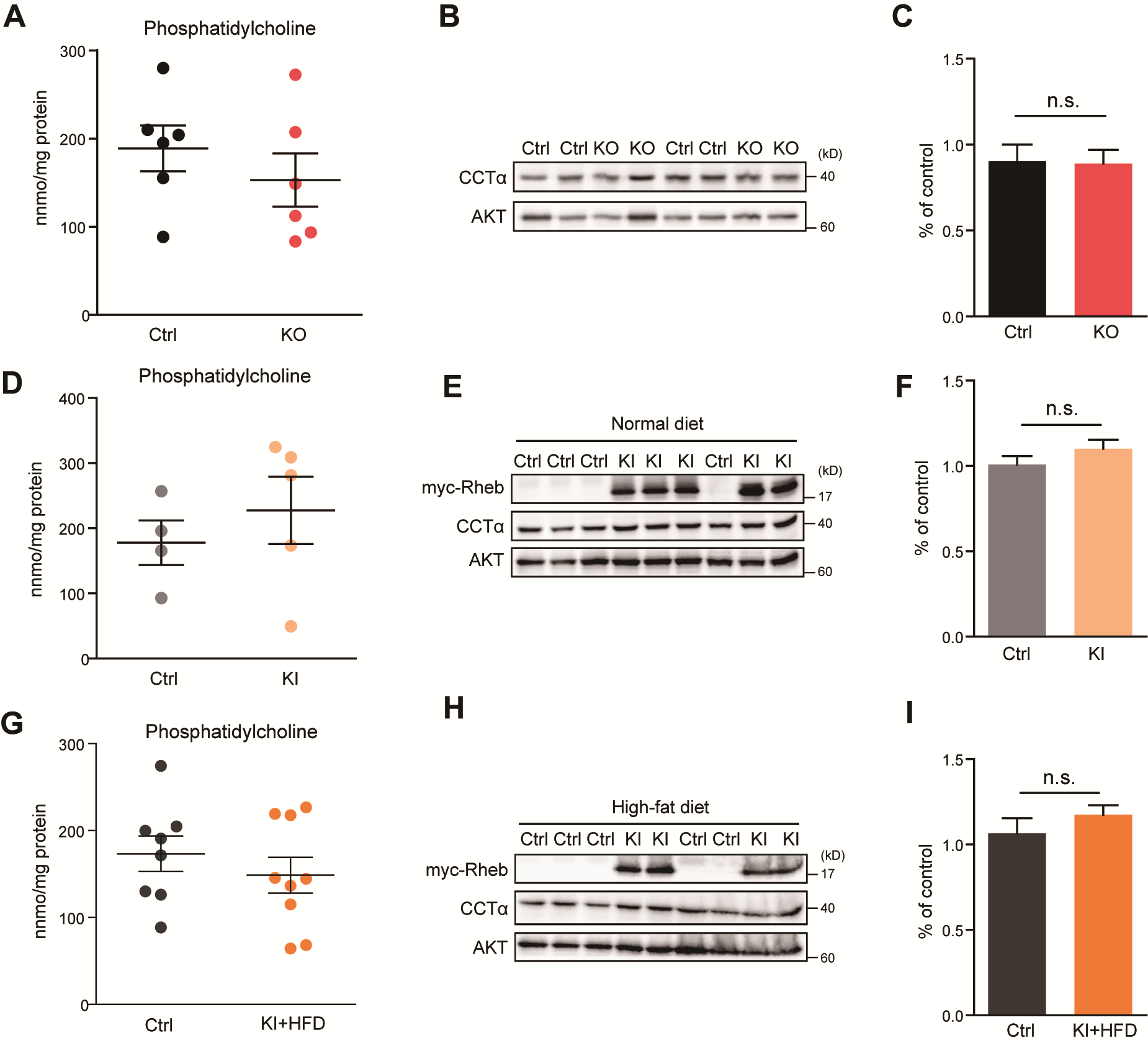


**(A)** The content of PC in the liver of *Rheb* KO. **(B-C)** Western blots **(B)** and quantifications **(C)** showing the protein level of CCTα in the liver of *Rheb* KO. **(D)** The content of PC in the liver of *Rheb S16H* transgenic (normal diet) mice. **(E-F)** Western blots **(E)** and quantifications **(F)** shows the protein level of CCTα in the liver of *Rheb S16H* transgenic (normal diet) mice. **(G)** The content of PC in the liver of *Rheb S16H* transgenic (HFD) mice. **(H-I)** Western blots **(H)** and quantifications **(I)** showing the protein level of CCTα in the liver of *Rheb S16H* transgenic (HFD) mice. Data represent mean ± SEM. n.s., no statistical significance.

**Supplementary Figure 10. FGF21 in the serum of *Rheb* KO and KI.**


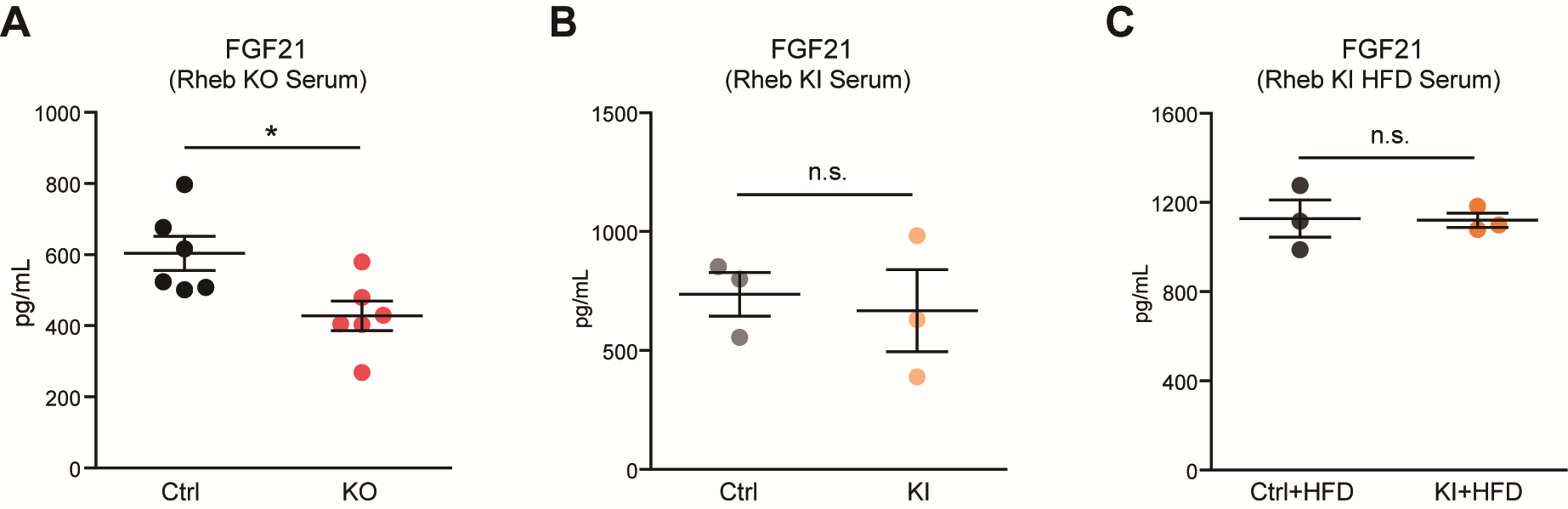


**(A)** FGF21 decreased in the serum of *Rheb* KO. (**B**) FGF21 does not change in the serum of *Rheb S16H* transgenic (normal diet) mice. **(C)** FGF21 does not change in the serum of *Rheb S16H* transgenic (HFD). Data represent mean ± SEM. **p*<0.05. n.s., no statistical significance.
